# α-Cyperone Alleviates LPS-Induced Pyroptosis in Rat Aortic Endothelial Cells via the PI3K/AKT Signaling Pathway

**DOI:** 10.3390/ph18030303

**Published:** 2025-02-22

**Authors:** Shuanghui Liu, Yankun Zhang, Xiaoxia Liang, Lizi Yin, Changliang He

**Affiliations:** 1 Department of Basic Veterinary, College of Veterinary Medicine, Sichuan Agricultural University, Chengdu 611130, China; liushuanghui@stu.sicau.edu.cn (S.L.); zhangyankun@stu.sicau.edu.cn (Y.Z.); 2 Natural Medicine Research Center, College of Veterinary Medicine, Sichuan Agricultural University, Chengdu 611130, China; liangxiaoxia@sicau.edu.cn (X.L.); yinlizi@sicau.edu.cn (L.Y.); 3 Department of Pharmacy, College of Veterinary Medicine, Sichuan Agricultural University, Chengdu 611130, China; 4 Department of Clinical Veterinary, College of Veterinary Medicine, Sichuan Agricultural University, Chengdu 611130, China

**Keywords:** pyroptosis, α-cyperone, NLRP3, caspase-1, GSDMD

## Abstract

**Objective:** To investigate the effect and underlying mechanism of α-cyperone in inhibiting pyroptosis in rat aortic endothelial cells (RAECs). **Methods:** Molecular docking technology was used to predict the potential binding affinity of α-cyperone to pyroptosis-related proteins. A pyroptosis model was established in RAECs using rat serum containing 10% LPS, with α-cyperone administered as a preventive treatment for 9 h. Cell viability and membrane integrity were assessed using propidium iodide (PI) staining and the CCK-8 assay. The release of IL-1β and IL-18 was quantified by ELISA. Western blot and RT-qPCR were performed to evaluate the expression levels of NLRP3, ASC, caspase-1 p20, and N-GSDMD. Additionally, RNA sequencing analysis was conducted to identify differentially expressed genes related to pyroptosis in LPS-induced RAECs following α-cyperone treatment, and key differential genes were validated by Western blot. **Results:** Molecular docking analysis reveals that α-cyperone exhibits a strong binding affinity to pyroptosis-related targets. α-Cyperone significantly improves LPS-induced cell viability (*p* < 0.001), reduces IL-1β and IL-18 release (*p* < 0.001), and downregulates the mRNA and protein expression of NLRP3, ASC, caspase-1, and GSDMD (*p* < 0.001). RNA sequencing indicates that α-cyperone primarily modulates pyroptosis-related gene expression in RAECs through the PI3K/AKT signaling pathway. Western blot validation further confirmed that α-cyperone effectively inhibited the protein expression of phosphorylated and total PI3K and AKT in RAECs (*p* < 0.001). **Conclusions:** α-Cyperone significantly alleviates morphological damage in the RAEC pyroptosis model, suppresses the release of proinflammatory cytokines IL-1β and IL-18, and potentially inhibits NLRP3/caspase-1/GSDMD activation through the PI3K/AKT signaling pathway, thereby attenuating LPS-induced pyroptosis in RAECs.

## 1. Introduction

Pyroptosis is a form of programmed cell death linked to inflammation, and it is driven by proteins from the gasdermin family [1]. It plays a crucial role in the initiation and development of numerous diseases. During pyroptosis, cells swell and the cell membrane ruptures, leading to the formation of numerous bubble-like protrusions on the membrane surface prior to rupture. Although membrane blistering also occurs in apoptosis, the two processes are distinct. Apoptosis is a regulated form of cell death that does not involve membrane rupture, while pyroptosis is characterized by pro-inflammatory membrane rupture, creating small holes with diameters in the range of 1–2 μm. This rupture allows extracellular water to enter the cell, resulting in cell swelling and osmotic lysis. As a result, pro-inflammatory cytokines, like Interleukin-18 (IL-18) and Interleukin-1β (IL-1β), along with caspase-1, are released via the pores, attracting inflammatory cells and initiating a strong inflammatory response [2,3].

Pyroptosis mechanisms are primarily classified into two pathways: the classical pathway and the non-classical pathway [4]. The key difference between the two lies in their dependence on the activation of caspase-1. In the classical pathway, pyroptosis relies on the activation of caspase-1, which is predominantly activated by inflammasomes [5]. Among these, the NOD-like receptor pyrin domain-containing protein 3 (NLRP3) inflammasome has been extensively studied. Among them, the NLRP3 inflammasome, a protein complex composed of NLRP3-ASC-caspase 1, has been extensively studied, which is activated in response to various danger and stress signals [6]. When the body is exposed to endogenous and exogenous stimuli, pathogen-associated molecular patterns (PAMPs) and damage-associated molecular patterns (DAMPs) disrupt the immune defense [7]. Pattern recognition receptors (PRRs) located on the cell membrane promptly detect these damage signals, recruiting NLRP3, apoptosis-associated speck-like protein (ASC), and the precursor of cysteine protease 1 (pro-caspase-1) to form the NLRP3 inflammasome complex, leading to the activation of caspase-1 [8]. Once activated, caspase-1 promotes the conversion of pro-inflammatory cytokine precursors, specifically interleukin-1β (pro-IL-1β) and interleukin-18 (pro-IL-18), into their biologically active forms, IL-1β and IL-18. Additionally, it enhances the synthesis of GSDMD. GSDMD forms pores in the plasma membrane, approximately 1–2 μm in diameter, facilitating the release of pro-inflammatory cytokines, particularly IL-1β and IL-18, into the extracellular space, thereby exacerbating the inflammatory response [9]. Concurrently, the formation of these membrane pores disrupts the Na⁺ and K⁺ concentration gradients across the cell membrane, resulting in an influx of water molecules. This influx surpasses the cell’s compensatory capacity, leading to rapid swelling, lysis, and eventual cell death.

Similar to the classical pathway of pyroptosis, the effector protein in the non-classical pathway is also GSDMD [10]. However, in the non-classical pathway, cytoplasmic LPS activates the precursors of caspases-4/5/11. The enzymatically active forms of these caspases can directly cleave GSDMD into its active fragment, GSDMD-N. While caspases-4/5/11 do not cleave pro-IL-1β and pro-IL-18, they facilitate the maturation and secretion of these cytokines through the NLRP3 inflammasome pathway [11].

α-Cyperone is a sesquiterpene compound extracted from sedge plants, such as *Cyperus rotundus* L., with a variety of biological activities [12,13,14]. It has been shown to exert antimicrobial activity by disrupting cell membranes and membrane-mediated apoptosis pathways, effectively inhibiting inflammatory and antioxidant stress responses, and its anti-inflammatory, antitumor, antibacterial, and neuroprotective effects have attracted more and more attention [15]. Specifically, α-cyperone reduces LPS-induced inflammation in BV-2 cells by activating the Akt/Nrf2 pathway while inhibiting the NF-κB pathway [16]. It also significantly mitigates IL-1β-induced inflammation and extracellular matrix (ECM) degradation in mice chondrocytes by blocking the activation of NF-κB and MAPK signaling pathways [17]. It has also been demonstrated that α-cyperone inhibits the activation of microglia and the expression of neuroinflammatory cytokines (TNF-α, IL-6, IL-1β, iNOS, COX-2, and ROS) by activating and inhibiting the NF-κB signaling pathway, alleviating LPS-induced neuroinflammation and oxidative stress in a rat model of Parkinson’s disease and RAW 264.7 cells [18,19]. In addition, studies have found that α-cyperone can improve neuropathic pain by intervening in the norepinephrine pathway, and can also inhibit the activation of the ROS-mediated PI3K/AKT/mTOR signaling pathway by regulating the mitochondrial apoptosis pathway, and promote HeLa cells apoptosis in human cervical cancer [20,21]. Therefore, α-cyperone may effectively improve LPS-induced inflammatory responses by participating in the processes of inflammation, oxidative stress, and apoptosis. However, there are currently no studies investigating whether it can inhibit inflammatory responses by intervening in cell pyroptosis. An in-depth examination of the specific mechanisms by which α-cyperone intervenes in cell pyroptosis could provide insights for its potential therapeutic use in treating various dis-eases related to cell pyroptosis triggered by inflammation or oxidative stress.

## 2. Results

### 2.1. Analysis of Molecular Docking Results

The molecular docking results are summarized in Table 1. The docking simulations indicate that α-cyperone demonstrates strong binding activity with cell pyroptosis-related proteins, including caspase-1, NLRP3, ASC, and GSDMD, exhibiting binding energies below −5 kcal/mol. Additionally, the analysis of binding modes reveals that α-cyperone interacts with multiple pairs of residues within these target proteins, highlighting its potential for effective targeting of cell pyroptosis-related proteins.

### 2.2. Cytotoxic Effect of α-Cyperone on RAEC

**Figure 1:**
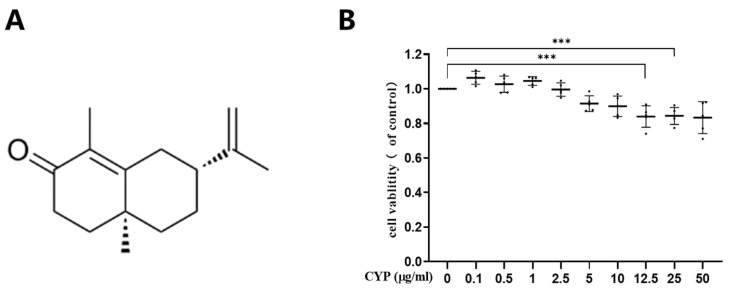
Cytotoxic effects of α-cyperone on RAEC cells. (**A**) Chemical structure of α-cyperone; (**B**) RAEC cells were treated with α-cyperone (0–50 μg/mL) for 18 h, and cell viability was assessed using the CCK-8 assay (n = 5). *** *p* < 0.001.

The cytotoxicity of α-cyperone on RAEC was assessed using the CCK-8 assay. The minimum and maximum doses for the toxicity test were set at 0.1 μg/mL and 50 μg/mL, respectively, with concentrations increased in a two-fold manner from 0.5 to 12.5 μg/mL over an 18 h exposure. As illustrated in Figure 1B, α-cyperone exhibits no cytotoxic effects within the concentration range of 0 to 10 μg/mL (*p* > 0.05).

### 2.3. α-Cyperone Can Alleviate LPS-Induced RAEC Cell Death

**Figure 2:**
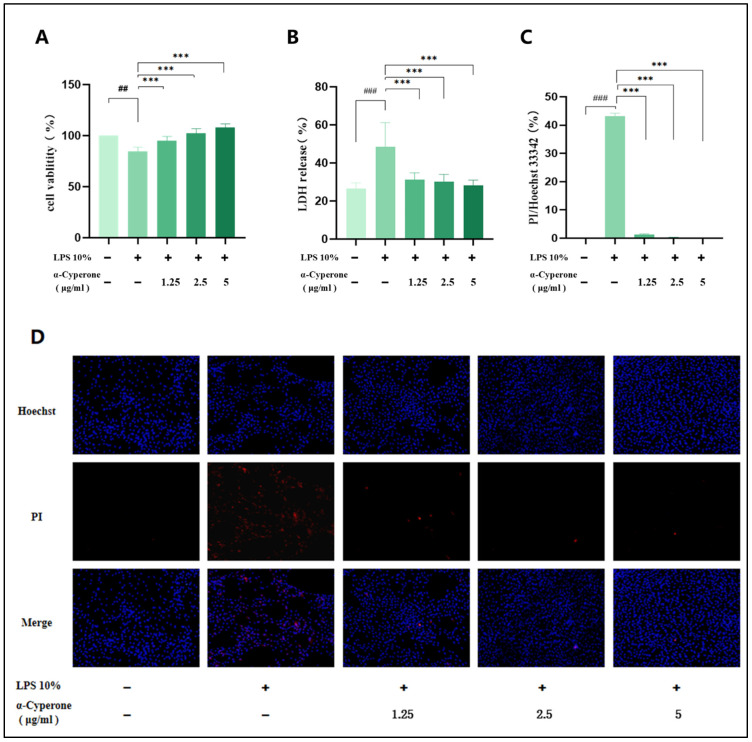
α-Cyperone attenuates LPS-induced cell mortality in RAEC cells. RAEC cells were treated with 10% LPS for 6 h, followed by pretreatment with or without α-cyperone (1.25, 2.5, and 5 μg/mL) for 3 h. (**A**) Cell viability assessed by the CCK-8 assay (n = 5); (**B**) cytotoxicity was determined by the lactate dehydrogenase (LDH) release assay (n = 5); (**C**) the rates of PI/Hoechst33342 staining in panel D were calculated. (**D**) Fluorescence microscopy images of Hoechst33342 and PI double staining (20×), merged with bright-field images. *** *p* < 0.001; ## *p* < 0.01, and ### *p* < 0.001.

To evaluate the impact of α-cyperone on LPS-induced RAEC cell death, cell viability was measured using the CCK-8 assay. The results in Figure 2A demonstrate that the cell viability of the LPS group 4 is significantly lower than that of the blank group (*p* < 0.001). Following α-cyperone pretreatment, cell viability was markedly increased (*p* < 0.001), indicating that α-cyronone effectively inhibits LPS-induced RAEC cell death.

LDH release and Hoechst 33342/PI double staining were employed to assess the effect of α-cyronone on LPS-induced membrane integrity. LDH release rates were measured, revealing that the LPS group exhibited significantly higher rates than the control group (*p* < 0.001). Following α-cyronone pretreatment, LDH release rates were dramatically reduced (*p* < 0.001). The results of Hoechst 33342/PI staining, shown in Figure 2C, indicate a significant increase in the ratio of membrane-damaged cells (PI: permeable, red) induced by LPS to total cells (Hoechst 33342, blue). After 3 h of α-cyronone pretreatment, the positive rate of PI staining was significantly decreased, consistent with the LDH results, confirming that α-cyronone significantly mitigates LPS-induced RAEC membrane damage.

### 2.4. α-Cyperone Inhibits LPS-Induced IL-1β/18 Release in RAEC Cells

**Figure 3:**
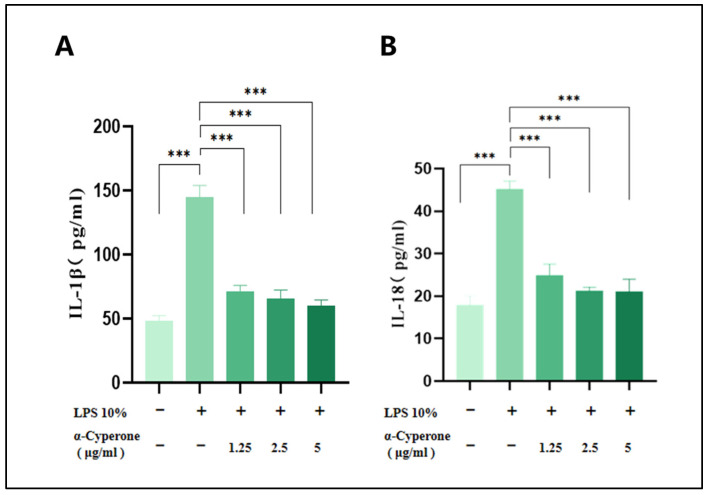
α-Cyperone inhibited the production of IL-1β and IL-18 in LPS-induced RAEC cells. The cells were treated with LPS10% for 6 h followed by pretreatment with α-cyperone (1.25, 2.5, and 5 μg/mL) for 3 h (**A**,**B**). The levels of IL-1β and IL-18 in the cell culture medium were measured by ELISA. Results are shown as mean ± SD (n = 8). *** *p* < 0.001.

IL-1β and IL-18 are critical pro-inflammatory cytokines cleaved by caspase-1 during pyroptosis. Decreasing the production of IL-1β and IL-18 can help mitigate various inflammatory responses triggered by LPS. The ELISA results reveal that α-cyperone (1.25–5 μg/mL) significantly reduces the levels of IL-1β and IL-18 in LPS-induced RAEC cells (Figure 3), demonstrating a clear dose-dependent effect.

### 2.5. α-Cyperone Inhibits the LPS-Induced Expression of Caspase-1/GSDMD/NLRP3/ASC in RAEC Cells

**Figure 4:**
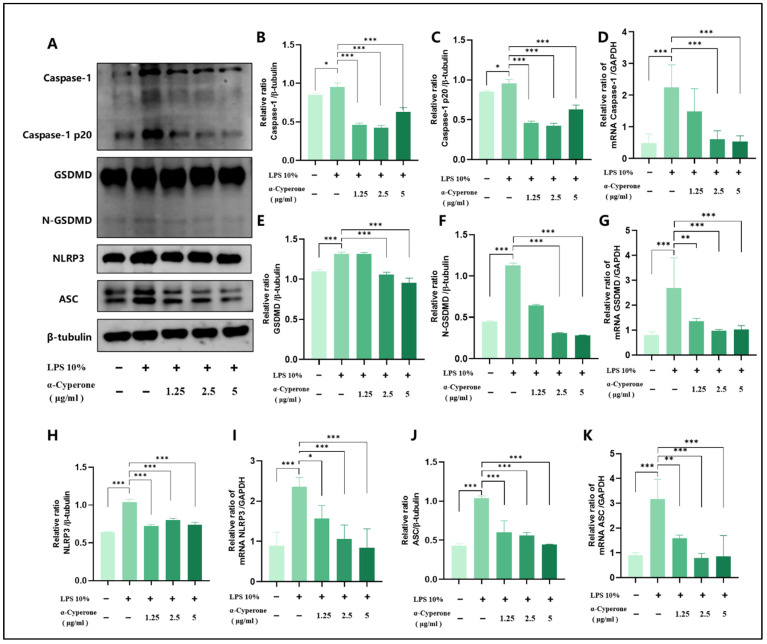
α-Cyperone alleviates LPS-induced pyroptosis in RAEC cells. (**A**–**C**,**E**,**F**,**H**,**J**) Western blotting detection of Caspase-1/Caspase-1 p20, GSDMD-N, NLRP3, and ASC in RAEC cells. (**D**,**G**,**I**,**K**) The mRNA levels of Caspase-1 GSDMD, NLRP3, and ASC were detected by qPCR. Results are shown as mean ± SD (n = 3). * *p* < 0.05, ** *p* < 0.01; *** *p* < 0.001.

RT-qPCR and Western blot analyses were performed to evaluate the expression levels of Caspase-1, GSDMD, NLRP3, and ASC, elucidating the mechanism by which α-cyperone acts in the LPS-induced pyroptosis of RAEC cells. The results show that LPS treatment significantly increases the expression levels of pyroptosis-related genes and proteins, including Caspase-1, GSDMD, NLRP3, and ASC (Figure 4). However, following α-cyperone intervention, the levels of these proteins were markedly reduced, confirming that α-cyperone inhibits LPS-induced RAEC pyroptosis via the Caspase-1/GSDMD/NLRP3 pathway.

### 2.6. Transcriptome Sequencing of RAEC Cells Induced by LPS Induced by α-Cyperone

To further investigate the impact of α-cyperone treatment on RAEC cell pyroptosis and its underlying molecular mechanisms, total RNA was extracted from control RAEC cells (control), LPS-treated RAEC cells (model), and LPS plus α-cyperone-treated RAEC cells (treatment) for RNA sequencing analysis, constructing an expression profile of RNA changes. Differentially expressed genes were identified based on log2 |Fold change| > 2 and *p* < 0.01. The results reveal that, compared to the control group, RAEC cells treated with 10% LPS exhibit 1998 differentially expressed genes (DEGs), with 905 genes upregulated and 1093 genes downregulated. When compared to LPS-treated RAEC cells, α-cyperone-treated RAEC cells exhibited 2318 DEGs, with 1596 genes upregulated and 722 genes downregulated.

**Figure 5:**
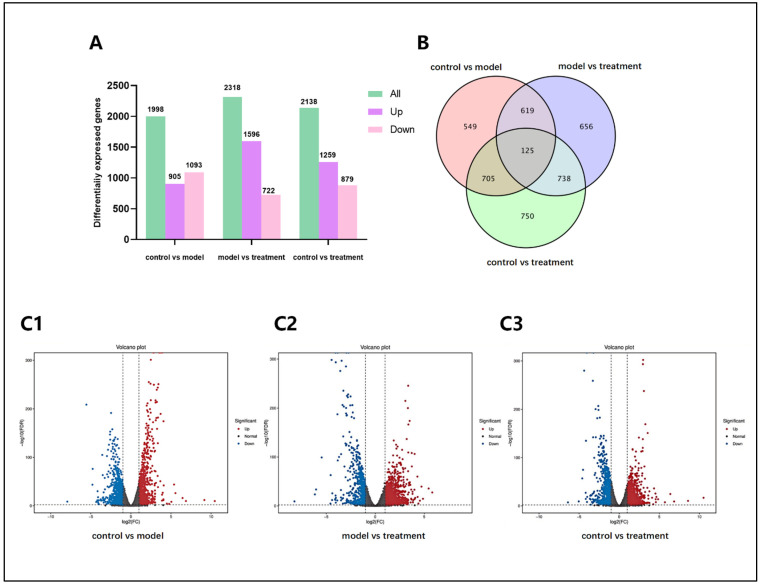
α-Cyperone treatment significantly altered the transcriptome profile of LPS-stimulated RAEC cells. Transcriptomes of control, LPS-stimulated RAEC cells, and α-cyperone-treated RAEC cells were constructed by RNA-seq. (**A**) Histogram of upregulated and downregulated genes. (**B**) Venn diagram of DEGs. (**C1**–**C3**) Volcano plots of DEGs from control, model (LPS stimulation), and treated (LPS plus α-cyperone treatment) RAEC cells.

Compared to the control group, α-cyperone-treated RAEC cells exhibited 2138 differentially expressed genes (DEGs), with 1259 genes upregulated and 879 genes downregulated (Figure 5A). There were 549 common DEGs between the control group RAEC cells and the model RAEC cells, 656 common DEGs between the model RAEC cells and the treatment RAEC cells, and 750 common DEGs between the control RAEC cells and the treatment RAEC cells; there were 125 common DEGs in the control, model, and treatment RAEC cells (Figure 5B). The volcano plot visually represents these results. Among the shared differentially expressed genes (DEGs), several were identified based on the comparison between the treatment and model groups, including CASP1, Pycard, NOD1, NOD2, IL18RAP, IL10RB, IL15, IL6, IL1α, TLR2, TLR3, TLR4, and TLR6. These genes are associated with the caspase family, NOD-like receptors, interleukins, and toll-like receptors. The findings suggest that α-cyperone downregulates the transcription and translation of these key genes, thereby influencing pyroptosis and inflammatory responses in RAEC cells.

### 2.7. DEG Profile and Functional Prediction Emphasize That α-Cyperone Inhibits LPS-Induced Pyroptosis in RAEC Cells via the PI3K/AKT Signaling Pathway

**Figure 6:**
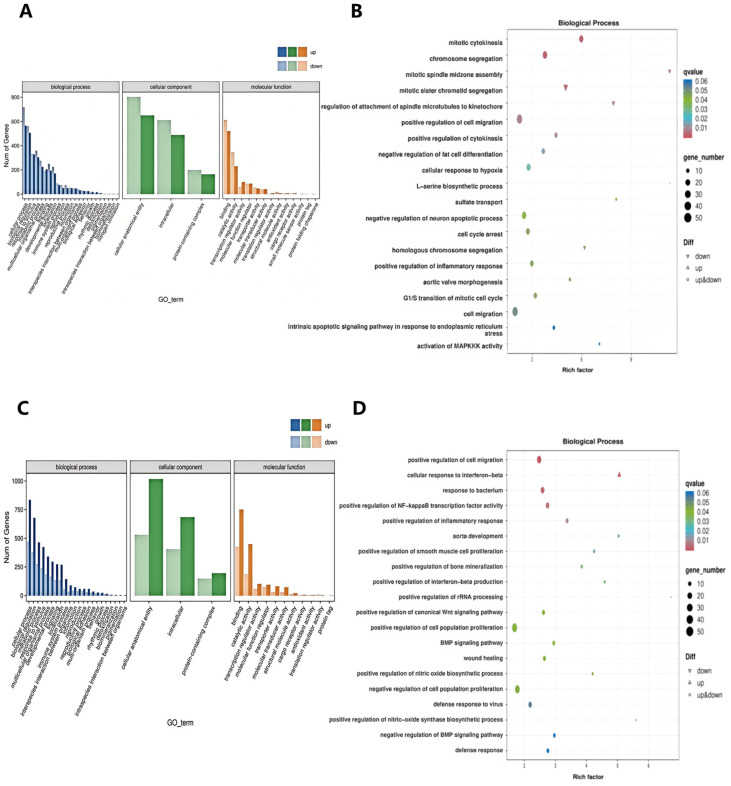
GO analysis of the differentially expressed genes (DEGs) revealed that α-cyperone inhibits LPS-induced pyroptosis in RAEC cells through the PI3K/AKT signaling pathway. (**A**,**B**): GO analysis of DEGs between the model and control groups. (**C**,**D**): GO analysis of DEGs between the treatment and model groups. The GO annotation of DEGs and the corresponding GO bubble plot display classifications of molecular function (MF), cellular component (CC), and biological process (BP). The color of the bubbles represents the Q-value, while the size of the bubbles indicates the number of differentially expressed genes within each functional category.

The functions of the aforementioned DEGs were characterized through Gene Ontology (GO) and Kyoto Encyclopedia of Genes and Genomes (KEGG) annotation-based enrichment analysis. The results of the GO enrichment analysis show that DEGs are mainly enriched in the biological process category. When RAEC cells treated with LPS10% were compared with control RAEC cells, DEGs were significantly involved in the regulation of cell migration, inflammatory response, endoplasmic reticulum stress, apoptosis, and MAPK signaling pathway activation (Figure 6A,B). Similarly, when RAEC cells treated with α-cyperone were compared with macrophages treated with LPS10%, DEGs were significantly involved in the cell response to IFN-β, aortic development, positive regulation of inflammatory response, and positive regulation of NO biosynthesis (Figure 6C,D).

**Figure 7:**
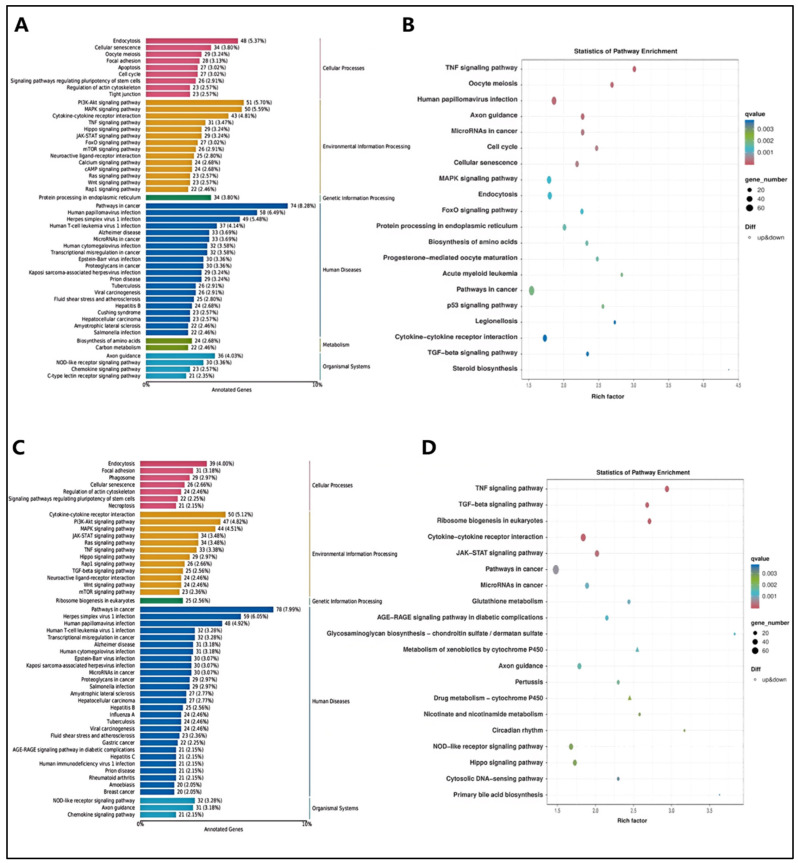
KEGG-annotated DEG enrichment analysis. (**A**,**B**): KEGG pathway classification diagram and DEG enrichment analysis comparing the model and control groups. (**C**,**D**): KEGG pathway classification diagram, and DEG enrichment analysis comparing the treatment and model groups. The KEGG pathway classification diagram and the KEGG bubble plot for DEGs are presented. In the bubble plot, the Q-value is represented by the color, and the size of the dots reflects the number of differentially expressed genes associated with each pathway.

To further explore the interactions between DEGs after α-cyperone treatment, we labeled and enriched the DEGs using the KEGG biological pathway database. In the KEGG analysis, compared with the control group, the DEGs in the model were highly enriched in the PI3K-AKT signaling pathway, cytokine–cytokine receptor interaction, MAPK signaling pathway, and TNF signaling pathway (Figure 7A,B). Interestingly, the DEGs between the treatment and model groups were notably enriched in the PI3K-AKT signaling pathway, cytokine–cytokine receptor interaction, MAPK signaling pathway, and TNF signaling pathway (Figure 7C,D). These findings suggest that α-cyperone may exert its anti-inflammatory and anti-pyroptosis effects mainly by inhibiting the cytokine–cytokine receptor interaction, as well as the PI3K-AKT, MAPK, and TNF signaling pathways.

### 2.8. α-Cyperone Inhibits the PI3K/AKT Signaling Pathway

**Figure 8:**
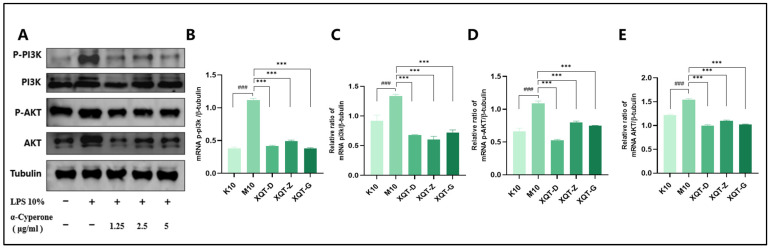
α-Cyperone can inhibit the protein expression of P-PI3K/PI3K/P-AKT/AKT induced by LPS in RAEC cells. (**A**–**E**) Western blotting detection of P-PI3K, PI3K, P-AKT, and AKT in RAEC cells. Results are shown as mean ± SD (n = 3). ### *p* < 0.001; *** *p* < 0.001.

To further confirm whether α-cyperone modulates cell pyroptosis through the PI3K/AKT signaling pathway, we analyzed the protein expression levels of PI3K and AKT. As shown in the Figure 8, after LPS10% stimulation, the expression of PI3K/AKT increases significantly, but after α-cyperone (1.25–5 μg/mL) intervention, the expression of PI3K/AKT decreases significantly, indicating that α-cyperone may affect cell pyroptosis by intervening in the activation of the PI3K/AKT signaling pathway.

## 3. Discussion

As a form of programmed cell death, pyroptosis serves as an innate immune defense response initiated by the body upon sensing infections from pathogenic microorganisms or endogenous danger signals [22,23,24]. It plays a crucial role in counteracting these stimuli and repairing damaged tissues. However, extensive research has demonstrated that pyroptosis exhibits a dual role. During disease progression, excessive pyroptosis can create a microenvironment conducive to disease development, severely jeopardizing the body’s health [25,26,27]. Thus, developing drugs that can inhibit pyroptosis is essential for preventing and treating various diseases [28].

Recent studies on pyroptosis have primarily focused on its mechanisms in macrophages and its involvement in different diseases. Notably, some research suggests that pyroptosis may act as an intrinsic factor in cardiovascular diseases, with gene-derived regulatory factors playing a significant role in their pathological processes [29,30,31]. Pyroptosis and its pathological byproducts can hinder vascular regeneration, damage vascular endothelial cells, destabilize arterial wall plaques, and contribute to myocardial hypertrophy and fibrosis, ultimately worsening disease outcomes and leading to poor prognoses [32,33,34,35]. Currently, most in vitro studies inducing endothelial cell pyroptosis in cardiovascular diseases utilize LPS [36,37]. However, inducing endothelial cell pyroptosis solely with LPS has proven challenging [38]. Consequently, we employed rat serum containing 10% LPS to create an endothelial cell-induced pyroptosis model in a rat aorta. The results indicate that cell viability significantly declines after stimulation with 10% LPS, leading to compromised cell membrane integrity. Furthermore, expressions of pyroptosis-related factors, such as Cleaved-Caspase-1, N-GSDMD, NLRP3, ASC, and IL-1β/IL-18, significantly increased, confirming that rat serum containing 10% LPS effectively induces strong pyroptosis in RAEC.

Many scholars have investigated the protective effects of natural drugs against pyroptosis and found that numerous traditional Chinese medicines aim to inhibit NLRP3 inflammasome activation and caspase-1 activity to treat cardiovascular diseases, regulating associated upstream pathway proteins, such as PI3K-AKT, AMPK, TLR4, or NF-κB [39,40,41,42]. However, the role and mechanisms of α-cyperone in modulating pyroptosis remain unexplored. Therefore, we first employed molecular docking technology to assess whether α-cyperone can bind to pyroptosis-related targets. The results indicate that α-cyperone binds effectively to multiple pyroptosis targets, including Caspase-1, GSDMD, NLRP3, and ASC, suggesting its potential as a drug for treating pyroptosis-related diseases. We then conducted in vitro experiments using α-cyperone to verify its ability to ameliorate LPS-induced pyroptosis in RAEC cells. Our data show that after α-cyperone intervention, cell viability is significantly enhanced with the increase in drug concentration, and the expression levels of pyroptosis-related proteins cleaved Caspase-1, N-GSDMD, NLRP3, and ASC in RAEC cells, and the release of IL-1β/IL-18 decreased in a dose-dependent manner, indicating that 5 μg/mL is likely to be the optimal dose of α-cyperone intervention for RAEC cell pyroptosis. This aligned with prior molecular docking results, confirming that α-cyperone can improve LPS-induced pyroptosis in RAEC cells via the Caspase-1/GSDMD/NLRP3 pathway.

In order to further understand the underlying molecular mechanism of α-carenone intervening in pyroptosis of RAEC cells, we continued to perform transcriptome analysis and screen for key genes that can regulate pyroptosis, and found that the PI3K-AKT signaling pathway may be a key target for α-carenone to regulate pyroptosis in RAEC cells. A large number of studies have shown that the PI3K-AKT pathway is one of the important signal transduction networks in cells, which can affect the occurrence of pyroptosis through multiple pathways. PI3K/AKT signaling may play different roles in different cell types and physiological conditions. In some cases, the overactivation of the PI3K/AKT pathway may lead to a cellular resistance to pyroptosis, thereby contributing to cell survival [43]. In other cases, the inhibition of the PI3K/AKT pathway may promote the inflammatory response and induce pyroptosis by activating the NLRP3 inflammasome and caspase-1. In this study, the expression of PI3K/AKT was significantly increased after LPS 10% stimulation of RAEC cells, while that of α-cianone was significantly decreased, suggesting that α-cimeketone is likely to further inhibit the pyroptosis of RAEC cells by inhibiting PI3K/AKT expression.

Pyroptosis can be used as a new target for the prevention and treatment of inflammatory diseases, and a variety of traditional Chinese medicine extracts and traditional Chinese medicine compounds can prevent and treat related diseases by inhibiting the mechanism of pyroptosis. However, the current mechanism between pyroptosis and related diseases is not thoroughly studied, and most of the existing studies are based on the regulation of NLRP3-related pyroptosis pathways, and there is a lack of studies on other proteins or pathways in the pyroptosis pathways. In this study, we only explored the idea that α-caramitone can inhibit the expression of PI3K, AKT, Caspase-1, GSDMD, and the activation of the NLRP3 inflammatory complex in vitro, and improve the LPS-induced pyroptosis of RAEC cells. In vivo studies on the intervention of α-cienketone in pyroptosis in RAEC cells through the PI3K-AKT signaling pathway and its specific mechanism need to be explored more deeply to clarify the complex relationship between this pathway and pyroptosis. At the same time, the possibility of involvement of other signaling pathways cannot be completely excluded. For instance, NF-κB, a classic pro-inflammatory pathway, has been shown to play a crucial role in the activation of NLRP3 inflammasomes. Additionally, AMPK, a key molecule in regulating cellular energy metabolism, is also implicated in the regulation of pyroptosis. Future studies should investigate whether α-cyperone exerts a synergistic or complementary effect through these pathways, which could offer a more comprehensive understanding of the underlying mechanisms.

## 4. Materials and Methods

### 4.1. Reagents

α-Cyperone (C_15_H_22_O, purity 98%) was purchased from Ainahua Chemical Reagent Co., Ltd. (Chengdu, China); LPS (L2880) was sourced from Sigma-Aldrich (Darmstadt, Germany). Anti-caspase-1, Anti-NLRP3, and Anti-β-tubulin antibodies were obtained from Proteintech (Wuhan, China); the Anti-GSDMD antibody from Abcam (Cambridge, UK); and the Anti-ASC antibody from MCE (South Brunswick, NJ, USA).

### 4.2. Molecular Docking

(1) Handling of Compounds

The 2D structure of α-cyperone was downloaded from the PubChem database (https://pubchem.ncbi.nlm.nih.gov/) (accessed on 28 July 2024) and subsequently converted into a low-energy 3D structure through energy minimization in MOE. This 3D structure was then prepared for molecular docking analysis using MOE.

(2) Screening of Protein Structures

The crystal structures of Caspase-1, GSDMD, NLRP3, and ASC proteins were retrieved from the RCSB Protein Data Bank (https://www.rcsb.org/) (accessed on 28 July 2024). The protein–ligand crystal complexes were selected with species set to human and resolution criteria of less than 3.0 A., and downloaded for further analysis.

(3) Molecular Docking

Molecular docking was carried out using a flexible induced-fit mode, which involved adjusting the side chains of the pocket amino acids to optimize the binding conformation. The weight of the constrained sidechain was set to 10 during rotation. The binding modes of the ligands were initially ranked using the London dG scoring function, and the top 30 conformations were further refined using the GBVI/WSA dG method to evaluate their binding free energies. The optimal binding mode was selected based on the maximum number of ligand–receptor binding sites. In cases where the number of binding sites was the same, the mode with the lowest binding energy was preferred. The binding pocket of the compound was defined by selecting the protein’s native ligand binding site. Finally, intermolecular binding patterns were analyzed and visualized using MOE 2019.0102 software.

### 4.3. Preparation of LPS Containing 10% Serum and α-Cyperone Drug Solution

Male Wistar rats (200 g) used in this study were provided by SiPeiFu Corporation (Beijing, China). Each rat received an intraperitoneal injection of a 0.5 mg/mL LPS solution at a dosage of 1 mL per 100 g of body weight. After 6 h, the rats were anesthetized, and cardiac blood was collected using a sterile disposable venous blood collection needle. Following centrifugation, the upper serum was carefully aspirated. The LPS rat serum was then mixed with a culture medium composed of 15% Australian fetal bovine serum (NEWZERUM), 100 U/mL penicillin, and 0.1 mg/mL streptomycin (HyClone, Logan, UT, USA) in high-glucose DMEM (BasaIMedia, Guangzhou, China), resulting in a final concentration of 10% LPS serum.

Dissolve 50 mg of α-cyperone in 5 mL of high-glucose DMEM culture medium containing 15% Australian fetal bovine serum, 100 U/mL penicillin, and 0.1 mg/mL streptomycin to obtain a 10 mg/mL α-cyperone drug solution. Then, dilute the solution to different concentrations of the α-cyperone drug solution using the high-glucose DMEM culture medium containing 15% Australian fetal bovine serum, 100 U/mL penicillin, and 0.1 mg/mL streptomycin according to the experimental needs.

### 4.4. Cell Culture and Treatment

Rat aortic endothelial cells (RAECs) were obtained from Xiamen Yimo Biotechnology Co., Ltd. (IM-R039, Xiamen, China) and cultured in high-glucose DMEM supplemented with 15% Australian fetal bovine serum, 100 U/mL penicillin, and 0.1 mg/mL streptomycin at 37 °C in a 5% CO_2_ environment. Following a 3 h pretreatment with α-cyperone, 10% serum containing LPS was added, and the cells were incubated for an additional 6 h.

Three independent experiments were conducted for each experimental group. For each experiment, cells within 20 generations were selected, and different cell batches were used to ensure the reliability of the results.

### 4.5. Cell Viability Assay

Cell viability was assessed using an enhanced cell counting kit-8 detection kit (Beyotime Biotechnology, Shanghai, China). RAEC cells were inoculated into 96-well plates (5 × 10^4^/well), and stimulation commenced when the cells reached approximately 80% confluence. After treating the cells with α-cyperone (1.25–5 μg/mL) at for 3 h, LPS-10% rat serum was added for an additional 6 h. After stimulation, the cell culture medium was replaced with 100 μL per well, followed by the addition of 10 μL CCK-8 reagent. The cells were incubated for 1 h, and the optical density (OD) value was read at 450 nm.

### 4.6. LDH Release Assay

The lactate dehydrogenase (LDH) cytotoxicity detection kit (Beyotime Biotechnology, Shanghai, China) was utilized to measure the LDH release rate from the cell culture supernatant. RAEC cells were seeded into 96-well plates and analyzed according to the kit’s instructions after the aforementioned treatments.

### 4.7. Hoechst 33342/PI Double Staining

The apoptosis fluorescence Hoechst 33342/PI double staining kit (Soleibao Science & Technology Co., Ltd., Beijing, China) was employed to detect the double staining of Hoechst 33342 and propidium iodide (PI).

RAEC cells were inoculated into a 12-well plate with a cell slide (2.5 × 10^5^/well), and stimulation began when cell growth reached approximately 80%. Following the same cell grouping and treatment as previously mentioned, cells were washed twice with pre-cooled PBS, then 800 μL of the staining buffer was added to each well. Subsequently, 4 μL of Hoechst 33342 and PI dye were added, and the cells were incubated at 4 °C in the dark for 20 min. After incubation, cells were washed twice with pre-cooled PBS to remove nonspecific staining, the liquid was aspirated, and the slide was placed on a slide with a drop of anti-fluorescence quencher for observation under a fluorescence microscope. Live cells appeared green, while dead cells appeared red.

### 4.8. Cytokine Detection

ELISA kits (Ruixin Biotech, Beijing, China) were employed to detect the concentrations of IL-1β and IL-18 in the cell culture supernatant for each group, following the manufacturer’s instructions.

### 4.9. RNA-Seq Analysis

Total RNA from each cell group was extracted following the TRIzol reagent protocol (Life Technologies, Carlsbad, CA, USA). Sequencing libraries were prepared using the Hieff NGS Ultima Dual-mode mRNA Library Prep Kit for Illumina (Yeasen Biotechnology, Shanghai, China), and sequencing was performed on the Illumina NovaSeq platform. Raw reads were processed using the BMKCloud bioinformatics pipeline tool (www.biocloud.net) (accessed on 14 November 2024). Differentially expressed genes (DEGs) were identified with DESeq2 (log2 |Fold change| > 2; *p* < 0.01), followed by Gene Ontology (GO) and Kyoto Encyclopedia of Genes and Genomes (KEGG) analysis.

### 4.10. Real-Time Reverse Transcription PCR

RAEC cells (3 × 10^6^/well) were seeded into T25 EasYFlask for the treatments described above. Total RNA was extracted using TRIReagent (Invitrogen, Grand Island, NE, USA). cDNA synthesis was performed with the TransScript^®^ All-in-One First-Strand cDNA Synthesis SuperMix for qPCR kit (Beijing, China), followed by real-time PCR analysis using iQ SYBR Green Supermix (Bio-Rad, Hercules, CA, USA). The mRNA levels of caspase-1, NLRP3, ASC, and GSDMD were measured using a CFX ConnectTM Real-Time PCR Detection System (Bio-Rad, Hercules, CA, USA). The primer sequences used in the study are provided in Table 2. All data were normalized to GAPDH mRNA expression levels.

### 4.11. Western Blot Detection

Total protein from RAEC cells in each group was extracted using RIPA lysis buffer (Cell Signaling Technology, Boston, MA, USA), and the protein concentration was determined with a BCA protein assay kit (Beyotime, Haimen, China). Equal protein amounts (20 μg) were loaded onto SDS-PAGE gels, with stacking gel run at 80 V for 30 min, and separation gel at 120 V for 1 h. The proteins were transferred to a membrane using wet transfer at 120 V, 200 mA for 35 min. The membrane was blocked with 5% skim milk powder for 1 h and incubated overnight at 4 °C with primary antibodies against caspase-1 (1:1000), NLRP3 (1:1000), ASC (1:1000), GSDMD (1:1000), and β-tubulin (1:5000). After washing three times with TBST for 5 min each, the membrane was incubated with a secondary antibody at room temperature for 1 h, followed by three additional washes with TBST. The membrane was then treated with an ECL luminescent solution (Bio-Rad, Hercules, CA, USA) for 1 min and imaged. The gray values of the bands were analyzed using Image J 1.52v software, and the results are expressed as the ratio of each group’s gray value to that of the blank group.

### 4.12. Results Analysis

All results are presented as mean ± SD, with the data obtained from three independent experiments. Statistical significance was assessed using analysis of variance (ANOVA; SPSS v22.0, IBM, Armonk, NY, USA), with *p* < 0.05 considered significant.

## 5. Conclusions

The application of 1.25–5 μg/mL α-cyperone significantly mitigates morphological damage in the RAEC cell pyroptosis model, markedly inhibits the release of the pro-inflammatory cytokine IL-1β/IL-18, and suppresses LPS-induced pyroptosis in RAEC cells through the NLRP3/caspase-1/GSDMD pathway. A total of 1.25–5 μg/mL α-cienone can significantly improve the morphological damage in the RAEC cell pyroptosis model, significantly inhibit the release of pro-inflammatory cytokine IL-1β/18, and may inhibit NLRP3/caspase-1/GSDMD activation through the PI3K/AKT signaling pathway. It also improves the LPS-induced pyroptosis of RAEC cells.

## Figures and Tables

**Table 1:** Molecular simulation of docking conformation and binding affinity.

Protein Name	2D Structure	3D Structure	Active Pocket	Binding Affinity (kcal/mol)
Caspase-1	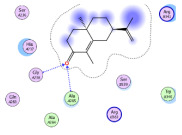	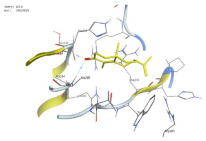	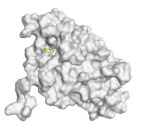	−5.285
GSDMD	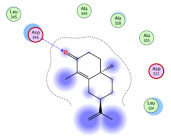	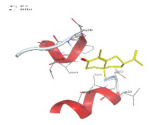	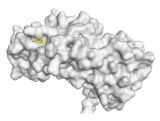	−5.348
NLRP3	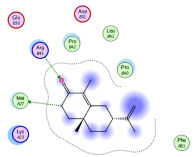	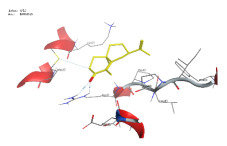	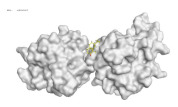	−5.047
ASC	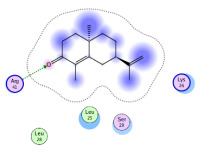	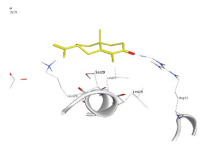	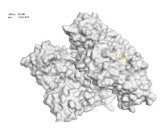	−5.160

**Table 2:** Primer sequences for RT-PCR.

Primer	Forward (5′→3′)	Reverse (5′→3′)
Caspase-1	CTGGGCAAAGGGAAGACTGTAGATG	ATGATGGCAACGATGGCAGGATAC
GSDMD	GTGAGCCACCCTGCTATTCA	GCAGGCATCCAGGCA ATAGA
NLRP3	GAGCTGGACCTCAGTGACAATGC	AGAACCAATGCGAGATCCTGACAAC
ASC	ATGGTTTGCTGGATGCTCTGTATGG	AAGGAACAAGTTCTTGCAGGTCAGG
GAPDH	ACTCTACCCACGGCAAGTTC	TGGGTTTCCCGTTGATGACC

## Data Availability

The original contributions presented in this study are included in the article. Further inquiries can be directed to the corresponding author.
